# The mysterious steps in carcinogenesis

**DOI:** 10.1038/sj.bjc.6605171

**Published:** 2009-07-28

**Authors:** D Brash, J Cairns

**Affiliations:** 1 Yale Comprehensive Cancer Center, Yale School of Medicine, New Haven, CT 06520-8040, USA; 2 Clinical Trial Service Unit, University of Oxford, Oxford OX3 7LF, UK; 3 105 Alleyn Park, London SE21 8AA, UK

The mutational changes needed to create a cancer cell have been itemised in great detail (Hahn and Weinberg, 2002). These mutations are commonly assumed to accumulate over the course of years as a consequence of spontaneous replication errors or, in special cases, the misrepair of DNA lesions introduced by carcinogens such as tobacco smoke or sunlight. Yet, there are numerous discrepancies between the mutagenicity of chemicals and their danger to humans (Clemmesen and Hjalgrim, 1977; Jansen *et al*, 1980; Ames *et al*, 1987), and the time course of carcinogenesis is deeply mysterious. Indeed, we still do not know the proximate causes of most cancers even though these are what we want to learn how to avoid.

The classical method for creating cancers, in rabbits (Friedewald and Rous, 1944) in mice (Berenblum and Shubik, 1947) and perhaps in humans (Pott, 1775), by applying coal tar to the skin, involves a sequence of steps, called initiation and promotion (Friedewald and Rous, 1944), which bear no obvious relation to what is now known about the molecular biology of cancer. For example, a mouse can be ‘initiated’ by feeding it once with 1 mg of the coal tar derivative 9,10-dimethyl-1,2-benzanthracene (Boutwell, 1964). This single treatment with a mutagen apparently causes cells to undergo a permanent change so that, for the rest of the mouse's life, its skin has become susceptible to the production of papillomas and carcinomas on exposure to the non-mutagenic irritants in croton oil. Surprisingly, a similar sequence has been seen using cells *in vitro*, where brief exposure to X-rays or methylcholanthrene apparently produces a permanent change in every cell so that, many generations later, its descendants occasionally undergo ‘spontaneous’ transformation into cancer cells (Kennedy *et al*, 1980, 1984). Why does there have to be a long interval between a cell's exposure to a mutagen and the expression of the resulting mutations, and why do only a minority of the cell's descendants express these mutations?

It would have been tempting to postulate that the various methods for producing cancer in the cells of animals are not good models of human carcinogenesis, were it not for the fact that humans and animals show the same strange relationship between dose of carcinogen and time of appearance of their cancers. For example, although the incidence of lung cancer in smokers appears to be directly proportional to the number of cigarettes smoked per day (Zaridze and Peto, 1986), it is proportional to roughly the sixth power of the duration of smoking. Similarly, when rats are continuously exposed to dietary carcinogens, their incidence of cancer rises as the first or second power of the dose rate but as a much higher power of time (Druckrey, 1967; Peto *et al*, 1997). If the carcinogen had simply to mutate a set of N genes to create cancer, the frequency of cancer should rise as the Nth power of the dose, and time would not be a major factor. These numerous experiments suggest, therefore, that mutagenic carcinogens cause just one or two events and that these (similar to the initial event in *in vitro* carcinogenesis) are then followed by steps that accumulate solely with the passage of time, driven perhaps by cell division. Thus, the rabbit whose ears have been painted with coal tar tends to develop its skin cancers where, months later, samples of its skin were excised and therefore had to be replaced by extra division in the surrounding epithelium (Linell, 1947). To take two well-documented examples of carcinogenesis in humans, a chimney sweep did not get scrotal cancer until after puberty when he had grown too large to climb chimneys (Pott, 1775); and smokers keep their raised rate of lung cancer for many years after they have stopped smoking (Halpern *et al*, 1993).

To recapitulate, there are examples where the sequence changes found in cancer cells are the type known to be produced by the initiating carcinogen (Brash *et al*, 1991; Hsu *et al*, 1991; Hainaut and Pfeifer, 2001), but the study of cancer in humans and animals, overall, has produced a very confused picture. The techniques of modern molecular biology have documented the many defects of cancer cells, so there is by now a fairly clear understanding of the basis for the dangerous properties of cancer cells. However, the picture that emerges from the classical studies of the epidemiology of human cancers and of experimental carcinogenesis in animals is hard to reconcile with what has been learnt about mutagenesis in simple systems such as the bacteria. Initiation seems to be far too efficient to be simply mutagenesis of certain oncogenes and suppressor genes, and the subsequent time-dependent steps are even more obscure.

In recent years, it has become plain that the management of biological information involves several unexpected mechanisms that limit the consequences when nucleic acids and proteins are damaged. Interactions between cells can protect against their individual defects (Rubin, 2006) and, on a larger scale, the expansion of abnormal clones can be inhibited by their normal neighbours (Chao *et al*, 2008). Within each individual cell, there is a large set of ‘heat-shock proteins’ (HSPs) that manages the folding and operation of the products of gene expression, and one of the actions of these HSPs is to sequester or remove the abnormal proteins produced as the result of mutation or chance misfolding. After exposure to harsh environmental conditions such as a sudden rise in temperature, the pattern of synthesis of the various HSPs is altered, and the inactivation of one of the many HSP genes has been shown to result in the expression of previously hidden mutations, suggesting that one function of HSPs is to allow complex evolutionary steps to occur under conditions of stress even when the changes entail a combination of several individually disadvantageous steps (Rutherford and Lindquist, 1998). Keeping those mutations masked may contribute to tumorigenesis, because inactivation of one of the HSP genes has been found to lower the frequency of skin cancer in mice exposed to initiation by dimethylbenzanthracene and promotion by phorbol esters (Dai *et al*, 2007).

The prime mystery in carcinogenesis remains the very first step, because it is hard to imagine how the numerous genetic changes found in cancer cells could have been produced in any cell as the result of a single exposure to a DNA-damaging agent, or why months or years should have to elapse before the effect of these changes is observed. Past speculations about the process of carcinogenesis (as opposed to the characteristics of the end product) have had little popularity and negligible impact. Indeed, the early suggestions that cancer cells owe their properties to mutation (Tyzzer, 1916) and that carcinogens interact with DNA (Boyland, 1952) were, in their time, violently attacked (Miller and Miller, 1952; Rous, 1959). Now, perhaps with all these recent discoveries of additional mechanisms that protect cells from damage, it may soon be possible to produce a plausible model for what is going on during carcinogenesis. At least one new idea seems to be needed, and the main purpose of this article is to alert readers to new developments that could at last start to clarify what is going on during carcinogenesis.
